# An Overview of National Alcohol Screening Day: Trends From 2001 to 2003

**Published:** 2004

**Authors:** Matthew E. Dupre, Robert H. Aseltine, Gene V. Wallenstein, Douglas G. Jacobs

**Affiliations:** Matthew E. Dupre, M.A., is a Ph.D. candidate in the Department of Sociology, Duke University, Durham, North Carolina. Robert H. Aseltine, Jr., Ph.D., is an associate professor in the Department of Behavioral Sciences and Community Health, University of Connecticut Health Center, Farmington, Connecticut. Gene V. Wallenstein, Ph.D., was formerly research manager at Screening for Mental Health, Inc., Wellesley Hills, Massachusetts, and currently is senior scientist at QualityMetric, Inc., Lincoln, Rhode Island. Douglas G. Jacobs, M.D., is executive director at Screening for Mental Health, Inc., Wellesley Hills, Massachusetts, and associate clinical professor, Department of Psychiatry, Harvard Medical School, Boston, Massachusetts

National Alcohol Screening Day (NASD) is the nation’s largest and most visible community-based intervention targeting alcohol misuse. Established in 1999 through a partnership between the National Institute on Alcohol Abuse and Alcoholism (NIAAA) and the Substance Abuse and Mental Health Services Administration (SAMHSA), and with support from numerous other public and private organizations, NASD has three primary objectives:

- To administer free and anonymous alcohol screening in settings accessible to the general public
- To provide referrals for treatment to those whom the screen identifies as drinking at unhealthy levels
- To educate the public on the impact of alcohol on general health.

Drawing on program data from 2001, 2002, and 2003, this sidebar offers a brief overview of the feasibility, sustainability, and efficacy of NASD.

## National Alcohol Screening Day: Background and Implementation

NASD takes place in April of each year. In preparation for the event, colleges, health care facilities, and community organizations across the country are recruited to take part in the program. Participating organizations receive materials that help them conduct the program, such as detailed instructions for setting up a site; educational handouts, posters, and videos; publicity materials such as news releases and prewritten op-ed pieces to submit to local media; and screening forms with specific instructions on how to administer and score them (see Greenfield et al. 1999, 2003 for program details). The NASD office, which is located in Wellesley Hills, Massachusetts, directs a national publicity campaign and hosts a Web site that offers further details.

On National Alcohol Screening Day, people stopping by the college and community sites are encouraged to complete the Alcohol Use Disorders Identification Test (AUDIT), a 10-question screening scale developed to identify those who consume alcohol at hazardous or harmful levels (see the description of common screening instruments on page 28 in this issue). A qualified health professional is available at most sites to score the responses, and people who score 8 or higher on the AUDIT are referred for further evaluation or treatment (Babor et al. 2001). Those taking the screen also may request to meet with a health specialist to discuss additional information on local resources (e.g., treatment centers, substance abuse counseling, and Alcoholics Anonymous meeting locations). Following the event, participating sites return the completed screening forms to NASD organizers so they can be encoded for statistical analysis.

## NASD Participation: Trends Over Time

The past 3 years have seen dramatic increases both in the number of sites registering for the program and the number of participants who visit each site. As table 1 indicates, 567 sites registered in 2001, 1,589 in 2002, and 2,621 in 2003, an almost fivefold increase. Based on data from the screening forms, the average number of participants screened per site increased from approximately 36 in 2001 to almost 52 in 2003, a 45-percent increase. Despite a notable growth in the program at college sites, the largest increases in participation occurred at sites such as hospitals, shopping malls, substance abuse clinics, and community centers, which saw an average increase of 156 percent in the number of participants per site from 2001 to 2003.

Table 1 data also indicate that increases in attendance at the typical NASD site have been accompanied by a greater clinician presence. From 2001 to 2003, the percentage of screening forms administered or reviewed by a health care professional at community sites (e.g., psychologist, psychiatrist, clinical social worker) increased by more than 52 percent, which means that more than 81 percent of screeners had clinical oversight in 2003, compared with 65 percent in 2001. Similar increases were observed at college sites, with 73 percent of screening forms reviewed by a clinician in 2003 compared with 60 percent of screening forms in 2001.

## Results

People who visit college screening sites and those visiting community sites differ markedly in some demographic variables, as table 2 demonstrates.

### College Findings

The profile of college attendees remained relatively stable between 2001 and 2003, despite the growth of the program. Slightly more than half of college participants were women (ranging from 54.7 to 56.2 percent), and as expected, well over 80 percent of participants were between the ages of 18 and 24. Approximately 67 percent of the total number of college participants for 2001 through 2003 were White, 16 percent were Black, and another 7 percent were categorized as Hispanic/Latino.

College participants’ AUDIT scores in each year, also presented in table 2, indicate that NASD identified a fairly substantial number of problem drinkers. Between 2001 and 2003, approximately 28 percent to 34 percent of all college participants’ AUDIT scores fell in the “hazardous” range (between 8 and 19) or the “harmful” range (20 and higher). The vast majority of those scoring over 8 on the AUDIT did not exceed the 20+ cutoff point that can indicate possible alcohol dependence. Rather, heavy episodic drinking, or “bingeing,” is the typical pattern for alcohol misuse among college students, a finding that has been substantiated in the literature (Wechsler et al. 1994).

### Community Site Findings

The demographic characteristics of people visiting community Screening Day locations also have remained fairly consistent despite the overall increase in NASD participation in recent years. Slightly more than half have been women, ranging from a low of 50.7 percent in 2001 to just over 55 percent in 2002. Approximately half were under the age of 35 in 2001, with this proportion increasing to almost 60 percent in 2003. In terms of race and ethnicity, table 2 shows that community sites generally attracted fewer Whites and substantially more Hispanic participants than did college sites, and the numbers of Asian and Native American participants increased.

As shown in table 2, NASD community participants’ AUDIT scores in the “hazardous” or “harmful” range increased from 20.8 percent in 2001 to 26.1 percent in 2003. Although the proportion of participants with AUDIT scores between 8 and 19 was consistently lower at community sites relative to colleges, in each year the proportion exceeding 19 was approximately 50 percent higher at community sites than at colleges.

### Limitations

One potential weakness of these data may be reflected in the declining proportion of registered sites returning screening forms each year, from 69 percent in 2001 and 54 percent in 2002, to 32 percent in 2003. Rather than being a compliance problem, however, further analysis suggests this observation was an artifact of program growth among the existing sites. Many of the most successful NASD sites expanded their programs to incorporate other locations. Although these satellite locations were listed as separate sites, their screening forms were submitted with those of the principal sites, making it appear that the number of sites was increasing but that the number of sites submitting screening forms was declining. For the 2005 NASD, principal sites will be able to submit their satellite sites’ screening forms separately.

## Conclusion

National Alcohol Screening Day is the nation’s first large-scale community-based program designed to identify and refer for evaluation and treatment members of the general public who engage in risky drinking. Early research on NASD suggested that it was effective in contacting previously unreached segments of the population whose drinking patterns were identified as unhealthy (levels of risky drinking among participants were modestly higher in 2002 and 2003 than in prior years; see Greenfield et al. 2003), and this conclusion is strongly reinforced by data from the past 3 years. The program continues to grow, with dramatic increases in the number of participating sites and the number of attendees per site between 2001 and 2003. Furthermore, increasing levels of clinician involvement in 2002 and 2003 show that these health professionals support NASD, and their participation helps ensure the quality and fidelity of administering the screen. Thus, recent data indicate that NASD continues to be a viable and sustainable community-based intervention for reaching those engaged in risky drinking behavior.

## Figures and Tables

**Table 1:** Characteristics of National Alcohol Screening Day Sites: 2001–2003

	2001	2002	2003
**All Sites**			
Number of sites registered	567	1,589	2,621
Number of sites reporting data	391	865	851
Number of screening forms collected	13,833	36,918	44,071
Mean number of participants per site	35.7	42.8	51.8
Percent of forms reviewed by clinician	61.1	67.3	76.9
**College Sites**			
Number of sites registered	250	560	883
Number of sites reporting data	233	370	364
Number of screening forms collected	11,255	24,434	24,060
Mean number of participants per site	48.3	66.0	66.1
Percent of forms reviewed by clinician	60.2	63.7	73.0
**Community Sites**			
Number of sites registered	317	1,029	1,738
Number of sites reporting data	158	495	487
Number of screening forms collected	2,578	12,484	20,011
Mean number of participants per site	16.3	25.2	41.1
Percent of forms reviewed by clinician	65.2	74.4	81.5

**Table 2:** Characteristics of National Alcohol Screening Day Participants: 2001–2003

	College			Community		

	2001 (*N*=11,113)	2002 (*N*=24,051)	2003 (*N*=24,060)	2001 (*N*=2,566)	2002 (*N*=12,042)	2003 (*N*=19,700)
**Gender**						
Male	45.3%	44.7%	43.8%	49.3%	44.9%	45.9%
Female	54.7	55.3	56.2	50.7	55.1	54.1
	100	100	100	100	100	100
**Age**						
18–24	86.0%	86.9%	82.4%	28.1%	23.7%	38.6%
25–34	7.8	7.5	8.7	22.5	20.5	19.7
35–44	3.4	3.2	4.3	22.1	22.0	18.1
45–54	2.1	1.9	2.9	16.6	17.3	14.2
55–64	0.7	0.5	1.2	7.8	7.8	5.9
65 or older	0.1	0.3	0.4	4.0	8.7	3.5
	100	100	100	100	100	100
**Race/Ethnicity**						
White	66.6%	68.1%	66.1%	48.0%	47.7%	46.6%
Black/African American	14.9	16.5	17.8	22.4	26.0	23.5
Hispanic/Latino	7.3	6.5	8.8	17.3	16.1	17.6
Asian	3.5	3.9	4.1	2.2	3.2	4.5
American Indian/Alaskan Native	1.1	0.7	0.9	2.9	4.8	5.4
Native Hawaiian/Pacific Islander*	—	—	0.5	—	—	0.7
Other	6.6	4.2	1.8	7.2	2.3	1.7
	100	100	100	100	100	100
**AUDIT Scores**						
No problem (<8)	71.6%	66.0%	67.6%	79.2%	75.8%	73.9%
Hazardous (8–19)	24.3	29.5	27.9	14.4	17.4	19.4
Harmful (20+)	4.1	4.5	4.5	6.4	6.8	6.7
	100	100	100	100	100	100

* This category was added in 2003.
